# Regulation of Sox2 and stemness by nicotine and electronic-cigarettes in non-small cell lung cancer

**DOI:** 10.1186/s12943-018-0901-2

**Published:** 2018-10-15

**Authors:** Courtney M Schaal, Namrata Bora-Singhal, Durairaj Mohan Kumar, Srikumar P Chellappan

**Affiliations:** 1 Department of Tumor Biology, H. Lee Moffitt Cancer Center and Research Institute, 12902 USF Magnolia Drive, Tampa, FL 33612 USA; 2 The Cancer Biology Ph.D. Program, University of South Florida, Tampa, FL USA

**Keywords:** Electronic cigarettes, Nicotine, Sox2, Cancer stem cell, Non-small cell lung cancer

## Abstract

**Background:**

Lung cancer is the leading cause of cancer related deaths and its incidence is highly correlated with cigarette smoking. Nicotine, the addictive component of tobacco smoke, cannot initiate tumors, but can promote proliferation, migration, and invasion of cells in vitro and promote tumor growth and metastasis in vivo. This nicotine-mediated tumor promotion is facilitated through the activation of nicotinic acetylcholine receptors (nAChRs), specifically the α7 subunit. More recently, nicotine has been implicated in promoting self-renewal of stem-like side-population cells from lung cancers. This subpopulation of cancer stem-like cells has been implicated in tumor initiation, generation of the heterogeneous tumor population, metastasis, dormancy, and drug resistance. Here we describe the molecular events driving nicotine and e-cigarette extract mediated stimulation of self-renewal of stem-like cells from non-small cell lung cancer.

**Methods:**

Experiments were conducted using A549 and H1650 non-small cell lung cancer cell lines and human mesenchymal stem cells according to protocols described in this paper. 2 μM nicotine or e-cigarette extracts was used in all relevant experiments. Biochemical analysis using western blotting, transient transfections, RT-PCR and cell biological analysis using double immunofluorescence and confocal microscopy, as well as proximity ligation assays were conducted.

**Results:**

Here we demonstrate that nicotine can induce the expression of embryonic stem cell factor Sox2, which is indispensable for self-renewal and maintenance of stem cell properties in non-small cell lung adenocarcinoma (NSCLC) cells. We further demonstrate that this occurs through a nAChR-Yap1-E2F1 signaling axis downstream of Src and Yes kinases. Our data suggests Oct4 may also play a role in this process. Over the past few years, electronic cigarettes (e-cigarettes) have been promoted as healthier alternatives to traditional cigarette smoking as they do not contain tobacco; however, they do still contain nicotine. Hence we have investigated whether e-cigarette extracts can enhance tumor promoting properties similar to nicotine; we find that they can induce expression of Sox2 as well as mesenchymal markers and enhance migration and stemness of NSCLC cells.

**Conclusions:**

Our findings shed light on novel molecular mechanisms underlying the pathophysiology of smoking-related lung cancer in the context of cancer stem cell populations, and reveal new pathways involved that could potentially be exploited therapeutically.

**Electronic supplementary material:**

The online version of this article (10.1186/s12943-018-0901-2) contains supplementary material, which is available to authorized users.

## Background

Despite growing insights into the mutational events that drive the genesis of NSCLC and the development of novel therapeutic strategies, lung cancer remains the leading cause of cancer related deaths. Lung cancer accounts for more deaths than breast, prostate, and colon cancers combined [1, 2]. Nicotine is the major addictive component of tobacco smoke; while it is not a carcinogen and cannot initiate tumors itself, nicotine has been shown to possess a number of tumor promoting properties in multiple tumor types, both in vitro and in vivo [3–9]. Nicotine exerts its tumor promoting functions through the activation of nicotinic acetylcholine receptors (nAChRs), which are typically expressed on neuronal cells; they are also expressed on cells of endothelial and epithelial origin, including tumor cells [10–12]. Our lab and others have shown that nicotine can promote proliferation, angiogenesis, epithelial-to-mesenchymal transition (EMT), migration, invasion, and survival of cultured non-small cell lung cancer cells. In addition, nicotine could also promote the growth and metastasis of lung and pancreatic cancers in mouse xenograft models, primarily through the α7 subunit of nAChRs [4, 5, 9, 13–15]. More recently, we have reported that nicotine can enhance the self-renewal of a subset of lung adenocarcinoma cells enriched in stem-like cell populations, through the induction of c-Kit ligand/Stem Cell Factor (SCF). SCF is known to promote self-renewal and differentiation of multiple stem cell types through the binding of its receptor, c-Kit [16–19], and this finding reveals a novel mechanism by which nicotine might be promoting tumor progression.

Tumors were traditionally thought to be a disease of clonal origin where a single transformed cell has the ability to give rise to heterogeneous tumor cell populations, with each daughter cell having the same capacity to give rise to more tumor cells. More recently, growing evidence supports the cancer stem cell model, which indicates that cancer stem-like cells (CSCs) arise through reprogramming of adult stem cells or progenitor cells, and these cells are responsible for tumor initiation, maintenance, progression and metastasis [20]; in addition, the tumor stem-like cells have also been shown to contribute to drug resistance, dormancy, recurrence, and metastasis [21, 22]. The model proposes that only CSCs are able to initiate tumors; these stem-like cells resemble traditional stem cells in that they are able to self-renew, divide asymmetrically, and are slow cycling [21]. Given these properties, understanding and targeting CSCs has become an important area of cancer research.

CSCs have been characterized and isolated using cell surface markers, which are differentially expressed on CSCs compared to non-stem-like, differentiated cancer cells. Various markers such as aldehyde dehydrogenase 1 (ALDH1) and CD133 positivity are effectively used for cancers such as breast, colon, brain, pancreas, head and neck [23–26]; however, there is no single marker ubiquitously expressed and used to identify lung cancer CSCs. A subset of tumor cells enriched in CSCs can be isolated based on their ability to efflux Hoechst 33342 dye out of their nuclei through the ABCG2 drug transporter expressed on the cell membrane of the stem-like cells, and have been termed as side-population cells, based on their distribution in flow cytometric sorting [27–29]. Our lab and others have shown that non-small cell lung cancer CSCs can be isolated using SP phenotype from cell lines as well as human tumor xenografts. Such cells were highly tumorigenic and produced highly invasive tumors in mice compared to the non-SP cells, and displayed stem-like properties such as the ability to self-renew, expression of epithelial-to-mesenchymal transition (EMT) markers as well as the classic embryonic stem cell transcription factors Sox2, Oct4, and Nanog [27, 30]. Sox2 itself was critical to maintain self-renewal of SP cells from NSCLC cell lines, compared to Oct4 and Nanog [27]. Since we find that Sox2 transcription factor is required to maintain NSCLC CSC stemness and that nicotine acts to enhance stemness, we sought to determine whether this nicotine-mediated promotion of stemness occurs through the induction of Sox2. Here we report that nicotine can induce Sox2 through a Yap1/E2F1/Oct4 signaling axis.

More recently, electronic cigarettes or e-cigarettes have been marketed as a healthy alternative to traditional cigarette smoking, as they do not contain tobacco, which contains multiple carcinogens such as polycyclic hydrocarbons, tobacco specific nitrosamines, and aldehydes [31, 32]. While e-cigarettes do not contain tobacco carcinogens, they contain nicotine in addition to other components such as propylene glycol, glycerol, and flavorings [32]. These devices are typically used by pressing a button which activates an internal heating coil which brings the e-liquid containing nicotine to a boil, which is then delivered as a vapor to the user [32]. The concentration of nicotine present in e-cigarettes varies by brand and container, but is typically represented as percent nicotine by volume (NBV). How nicotine present in e-cigarettes impacts the pathophysiology and health of users remains unclear, and whether the additional components of the e-liquid might abrogate or amplify the effects of nicotine in the context of cancer has not been determined. Here we report that e-cigarette extracts can promote self-renewal in a manner similar to nicotine; further, e-cigarette extracts could induce Sox2 expression, suggesting that exposure to nicotine, either through tobacco smoke or through the use of e-cigarettes, might have deleterious effects.

## Methods

### Cell lines

Human non-small cell lung adenocarcinoma cell lines A549 and H1650 were obtained from the American Type Culture Collection (ATCC). A549 cells were maintained in Ham’s F12K medium (Cellgro, Mediatech, Inc.) supplemented with 10% fetal bovine serum (Atlas Biologicals), and H1650 cells were maintained in RPMI 1640 (Gibco, Life Technologies, Thermo Fisher Scientific Inc.) containing 10% fetal bovine serum. Normal human bone marrow derived mesenchymal stem cells (hMSCs) were purchased from Lonza and maintained in their mesenchymal stem cell basal growth medium (MSCGM) designed to maintain these cells in a proliferative but not differentiated state. A549 and H1650 cell lines have been validated by ATCC and were validated again on May 25, 2016. hMSCs were pre-validated by Lonza, and only used upto passage 10.

### Generation of stable cell lines

A549 cell line was used for generating stable overexpression cells. The Sox2-core-luc (Bora-Singhal et al., 2015) and YAP1 (Addgene #15682), Oct4 (Addgene #17964) [33] and E2F1 expression vectors [13] were transfected using FugeneHD reagent (Promega) per manufacturer’s protocol. The transfected cells were selected using G418 and puromycin and maintained in Ham’s F12 K medium; single colonies were selected and expanded for use in experiments.

### Nicotine, E-cigarettes, and inhibitor studies

(−)-nicotine (N3876; Sigma-Aldrich) or e-cigarettes (local stores) were used in these studies. A549 or H1650 cells were rendered quiescent by serum starvation in media containing 0.1% fetal bovine serum for 24 h, following which cells were stimulated with 2 μM nicotine or e-cigarette extracts for the indicated time points. For studies using nicotine or e-cigarette extracts in hMSCs, cells were maintained in stem cell media and stimulated with 2 μM nicotine or e-cigarette extracts 24 h after plating, for indicated time points. For studies using signal transduction inhibitors/anti-cancer drugs, cells were rendered quiescent by serum starvation for 24 h, were treated with inhibitors for 30 min prior to stimulation with 2 μM nicotine; the cells were maintained in the serum free medium during nicotine stimulation. The inhibitors used were AZD0530/Saracatinib (Sellekchem) at 10 μM, NVP-BKM120/Buparsilib (Chemietek) at 20 μM, GSK1120212/Trametinib (Chemietek) at 10 μM, LEE001/Ribociclib (Chemietek) at 20 μM, RRD251 at 10 μM, α-bungarotoxin (Sigma) at 10 μM, or visudyne (Sigma) at 2 μM.

Three different brands of e-cigarettes were used to demonstrate the effects; these included Fin, Njoy, and Mistic (which are referred to as E-cig 1, E-cig2, or E-cig3, respectively). E-cigarette liquid was obtained through extraction of an internal liquid-soaked sponge within the devices for E-cig 1 and 2, or by syringe extraction for E-cig 3. E-cig 1, 2, and extracts were 1.6% nicotine by volume (NBV) or 16 mg/ml, 1.5% NBV or 15 mg/ml, and 1.8% NBV or 18 mg/ml respectively as indicated on the manufacturer’s packaging. Molarity of extracts from each brand was calculated based on the molecular weight of nicotine of 162.23, and the working concentration of 2 μM was achieved by serial dilutions of 1:10, 1:9, or 1:11 for E-cig 1, 2, or 3 respectively, to achieve 10 mM, then diluted 1:50 for a final concentration of 2 μM.

### siRNAs and antibodies

siRNAs used were purchased from Santa Cruz Biotechnology including Oct3/4 (sc36123), TEF4/Tead2 (sc45232), α7 nAChR (sc42532), E2F1 (sc29297), c-Src (sc44250), Sox2 (sc38408), Yap1 (sc38637), c-Yes (sc29860), and β-arr-1 (sc29741). Antibodies used for western blot against Sox2 (3579s), p-Src (2101s), p-AKT (9018p), pan-AKT (C67E7), Oct4 (2750s), p-ERK1/2 (9101s), and total ERK1/2 (9102s) were purchased from Cell Signaling Technologies; against c-Src (05–184) from EMD Millipore; against E2F1 (sc251) from Santa Cruz Biotechnology; against Yap1 (53–161) from Abnova, α7 nAChR (ab23832 and ab10096) from Abcam; and Actin from Sigma Aldrich (A1978).

Antibodies used for chromatin immunoprecipitation (ChIP) assays included E2F1 (sc193), E2F2 (sc633), E2F3 (sc879), E2F4 (sc1082), E2F5 (sc999), Rb (sc50), from Santa Cruz Biotechnology; Yap1 (ab56701), H327Kme3 (ab9045), and H327Kme1 (ab6002) from Abcam; a rabbit anti-mouse secondary antibody from Pierce was used as a negative control.

Antibodies used for immunofluorescence included Sox2 (3579s) from Cell Signaling Technologies; ZO-1 (339100) from Invitrogen; E2F1 (sc193), Crm1 (sc5595), and E-cadherin (sc8426) from Santa Cruz Biotechnology; α7 nAChR (ab10096) Yap1 (ab56701) from Abcam.

### ChIP-PCR experiments

Chromatin immunoprecipitation assays were conducted using previously described protocols [34, 35]. Interactions of the proteins with specific regions of the Sox2 promoter were detected by PCR amplification using the following primer sequences:

F1–5’-GAAAAGGCGTGTGGTGTGAC3–3′;

R1–5′- CGCTGATTGGTCGCTAGAAAC -3′;

F2–5’-GGGAGTGCTGTGGATGAGC-3′;

R2–5’-GTGGGTAAACAGCACTAAGACTACGTG-3′;

F3–5’-TGTGCGCTGCCTGCACCTGTG-3′;

R3–5’-ACTCCAGCAGAACCAGCCCTG-3′;

F4–5’-ACGTGCTGCCATTGCCCTC-3′;

R4–5’-CGGGTTAGAGGAGGATGAGA-3′.

### Transient transfections and luciferase assays

Cells transfected in Opti-MEM medium (Gibco, Life Technologies) using Fugene HD (Promega) transfection reagent following the manufacturer’s protocol. The mutSox2-core-luc construct containing a mutated Oct4 binding site was previously generated using Quikchange Lightening multi-site-directed mutagenesis kit (Agilent Technologies), as previously reported from our lab [30]**.** To confirm the role of E2Fs in regulation of the Sox2 promoter, the 7 E2F consensus binding sites present in the 500 bp region upstream of TSS (where TSS = 0) were mutated. This was done by mutating each of the 4 bp CGCG consensus sites to AATT within the Sox2-core-promoter at the following positions: -37 bp through -40 bp, − 50 bp through -53 bp, − 107 bp though -110 bp, − 119 bp through -122 bp, − 336 bp through -339 bp, − 361 bp through -364 bp, and -476 bp through -479 bp. Mutation of the E2F sites was outsourced to Genscript USA, Inc., and the E2F-mutant Sox2-core promoter was then cloned into pGL3 expression vector by our lab, for use in transient transfection experiments. The expression vectors used were pcDNA3-HA-E2F1, pcDNA3-E2F2, pcDNA3-E2F3, pcDNA3-E2F4, pcDNA3-E2F5, and Yap1 (Addgene #18978). Empty vector pcDNA3 was used as a control. Luciferase assays were conducted 24 to 48 h after transfection per manufacturer’s protocol using the Dual Luciferase Assay system (Promega). Results are reported as relative luciferase activity (RLA) based on the ratio of RLUs1 (firefly luciferase) to RLUs2 (Renilla luciferase: normalization control) values as measured on a Turner Biosystems luminometer.

### siRNA transfections and quantitative real-time PCR

Cells were transfected in Opti-MEM (Gibco Life Technologies) with 100 pmol of siRNAs using Oligofectamine reagent (Invitrogen) as per the manufacturer’s protocol. Media was replaced by complete medium containing 10% FBS 4–6 h after transfection. RNA was isolated using Qiagen RNEasy miniprep kit (Hilden, Germany) according to manufacturer’s protocol. First strand cDNA was synthesized using Bio-Rad iScript cDNA synthesis kit (Hercules, CA). mRNA expression was assessed using qRT-PCR (Bio-Rad CFX96 Real Time System) and data were analyzed using the CFX96 software. RT-primers used were as follows:

GAPDH(F): 5’-GGTGGTCTCCTCTGACTTCAACA-3′;

GAPDH(R): 5’-GTTGCTGTAGCCAAATTCGTTGT-3′.

Vimentin(F): 5’-GGACCAGCTAACCAACGACA-3′;

Vimentin(R): 5’-AAGGTCAAGACGTGCCAGAG-3′;

Fibronectin(F): 5’-TAGATGTACAGGCTGACAGA-3′;

Fibronectin(R): 5’-TCTTTCTTAAGCCCTTTGCT-3′;

Yap1(F): 5′- CCCAAGACGGCCAACGTGCC-3′;

Yap1(R): 5′- ACTGGCCTGTCGGGAGTGGG-3′;

Sox2(F): 5′ – GGGAAATGGGAGGGGTGCAAAAGA-3′;

Sox2(R): 5′- TTGCGTGAGTGTGGATGGGATTGG-3′;

ZEB1(F): 5’-AGCAGTGAAAGAGAAGGGAATGC-3′;

ZEB1(R): 5’-GGTCCTCTTCAGGTGCCTCAG-3′;

ZEB2(F): 5’-ATCTGCTCAGAGTCCAATGCAGCAC-3′;

ZEB2(R): 5’-AACAGTATTGTCCACAATCTGTAG-3′.

Data was normalized using GAPDH as an internal control, and fold change was determined using the 2^-ΔΔCT^ method.

### Lysate preparation and IP/Western blotting

Cell lysates were prepared and processed for western blotting as described in our previous work [30, 35]. Protein was detected using ECL reagent from GE Healthcare or Pierce Biotechnology according to standard protocols; actin was used as a control.

For co-immunoprecipitation assays, 200 μg of total protein lysate from A549 and H1650 cells were incubated with 4 μg of indicated antibodies. An equal amount of non-specific IgG from rabbit or mouse serum (Sigma-Aldrich) was used as a negative control. The interacting proteins were detected by western blotting.

### Immunofluorescence analysis and confocal microscopy

Immunofluorescence assays were conducted as previously described [30, 36]. Cells were visualized with a DM16000 inverted Leica TCS SP5 tandem scanning confocal microscope at 630× or 1890× magnification.

### Proximity ligation assays (PLA)

PLA studies were conducted as previously described using Duolink assay system (Sigma-Aldrich) [14, 30, 36]. The images were taken using Leica TCS SP5 confocal microscope (Leica Microsystems) at  630x and  1890x magnification.

### Isolation of side-population (SP) cells and self-renewal

SP cells were isolated from heterogenous cell populations using flow cytometry based on Hoechst 33342 dye efflux, and were then plated for self-renewal assays on low-adherence plates in stem-cell selective media, using protocols described in detail earlier [27, 37]. For experiments involving nicotine or e-cigarette extracts, these were added directly into stem-cell media at the time of plating. For depletion experiments, cells were transfected using siRNA and SP cells were isolated 48 h later.

### Wound healing assays

Wound healing or scratch assays were conducted as previously described [4, 38]. Images were taken every 24 h for 48 h, using EVOS FL microscope system (Life Technologies) at 10× magnification.

### Statistical analysis

All data have been statistically analyzed using Microsoft Office Excel 2010 (Microsoft Corporation, Redmond, WA). The data presented here is with ± standard deviation (SD) values derived from three independent experiments unless otherwise stated. The statistical comparisons between the groups were carried out by unpaired two tailed Student’s t-test or one-way ANOVA to calculate the *p* value for statistical significance. *p < 0.05, **p < 0.01 and ***p < 0.001.

## Results

### Nicotine and e-cigarette extracts enhance self-renewal of SP cells and promote EMT

**Fig. 1:**
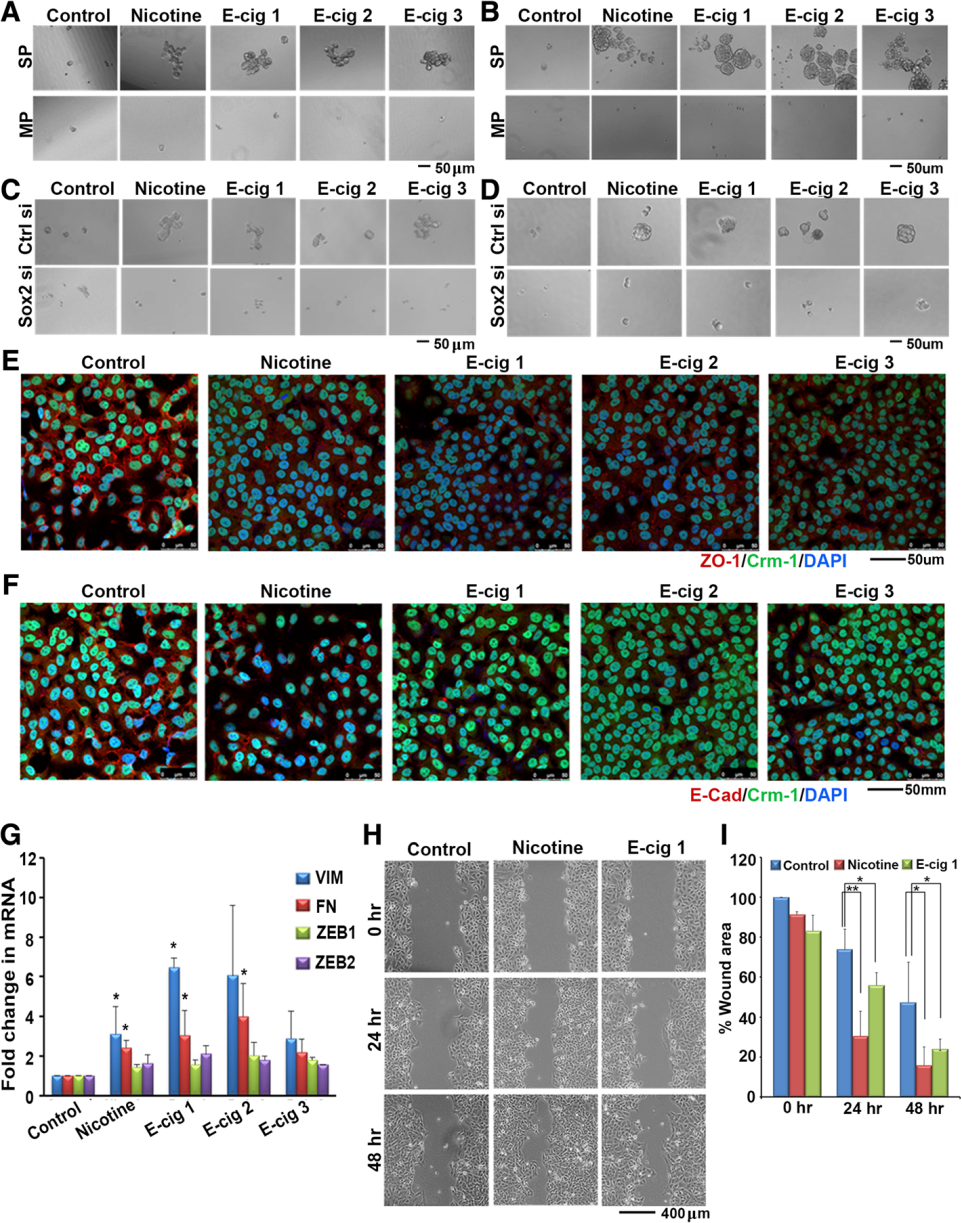
**a** Nicotine and E-cigarette extracts promote the self-renewal of stem-like SP cells from A549 cells; similar results were obtained on H1650 cells (**b**). SP represents side population and MP represents main population cells. Depletion of Sox2 using a siRNA abrogates the self-renewal of SP cells from A549 cells (**c**) or H1650 cells (**d**). Nicotine and E-cigarette extracts induce EMT-like changes in A549 cells. Immunofluorescence experiments using an antibody to ZO1 shows that nicotine and E-cigarette extracts reduce the tight junctions effectively (red fluorescence); CRM1 and DAPI are used to visualize the cells (**e**). Similar results were observed with E-cadherin (**f**). **g** A RT-PCR experiment shows the induction of mesenchymal markers vimentin and fibronectin as well as ZEB1 and ZEB2. The data presented here is with ± standard deviation (SD) values derived from three independent experiments. The statistical comparisons between the groups were carried out by unpaired two tailed Student’s t-test. **h-i** Nicotine and E-cigarette extracts promote the migration of A549 cells on plastic, as seen by a wound-healing assay (**h**) and the healing of the wound was represented graphically as % wound area over time (**i**). The statistical comparison between the groups was carried out by unpaired two tailed Student’s t-test

We had previously found that nicotine could enhance the self-renewal ability of SP cells, so we next determined whether this was observed with e-cigarette extracts. SP cells were isolated from A549 and H1650 cell lines by FACS, and SP and MP cells were plated separately on low adherence plates in stem cell selective media. Nicotine or extracts from each of the three brands of e-cigarettes was added to the media in the corresponding wells at the time of plating. E-Cigarette extracts were added equivalent to 2 μM nicotine, as described in the Materials and Methods. After 10 days cells were imaged to assess sphere formation as an indication of self-renewal using microscopy. It was found that e-cigarette extracts could enhance sphere formation and self-renewal in a manner similar to nicotine, in both A549 (Fig. 1a) and H1650 cells (Fig. 1b). Our earlier studies had demonstrated that Sox2 was necessary for the self-renewal of SP cells from lung adenocarcinoma cell lines. To examine if Sox2 was also needed for nicotine and E-cigarette mediated enhancement of self-renewal, Sox2 was depleted using siRNA. While transfection of a control siRNA did not affect the self-renewal of SP cells in response to nicotine or E-cigarette extracts, depletion of Sox2 abolished sphere formation in both the cell lines (Fig. 1c and d).

Epithelial-to-mesenchymal transition (EMT) is a normal process during development that is frequently activated during tumor progression allowing for a more migratory and invasive phenotype [39, 40]. Growing evidence suggests that activation of EMT induces the acquisition of stem cell properties in epithelial cells and that the induction of EMT and emergence of CSCs is strongly linked [39, 40]. We have previously reported that, in addition to enhancing CSC properties, nicotine can induce a number of factors involved in EMT including mesenchymal markers such as vimentin, fibronectin, Zeb1, and Zeb2; further, it could suppress epithelial markers such as E-cadherin and disrupt the tight junction protein ZO-1, thereby enhancing cell motility, cell migration and invasion [4, 13]. Since we also find e-cigarette extracts are capable of enhancing CSC properties, we next interrogated whether e-cigarette extracts could enhance EMT phenotypes, perhaps as a precursor to the acquisition of CSC phenotype. Immunofluorescence assays demonstrated that e-cigarette extracts could disrupt ZO-1 tight junction protein with a concordant decrease in ZO-1 staining overall, and could additionally reduce levels of E-cadherin in A549 cells (Fig. 1e and f; red fluorescence). At the mRNA level, we found that each of the three e-cigarette brands could induce the mesenchymal markers vimentin, fibronectin, Zeb1, and Zeb2 (Fig. 1g). Wound healing assays additionally demonstrated that e-cig 1 could enhance cell migration to a certain extent (Fig. 1h); the results are quantified in Fig. 1i. Overall, this data suggests that e-cigarette extracts induce EMT phenotypes in a manner similar to what was previously observed with nicotine.

### Nicotine and e-cigarette extracts enhance Sox2 expression

**Fig. 2:**
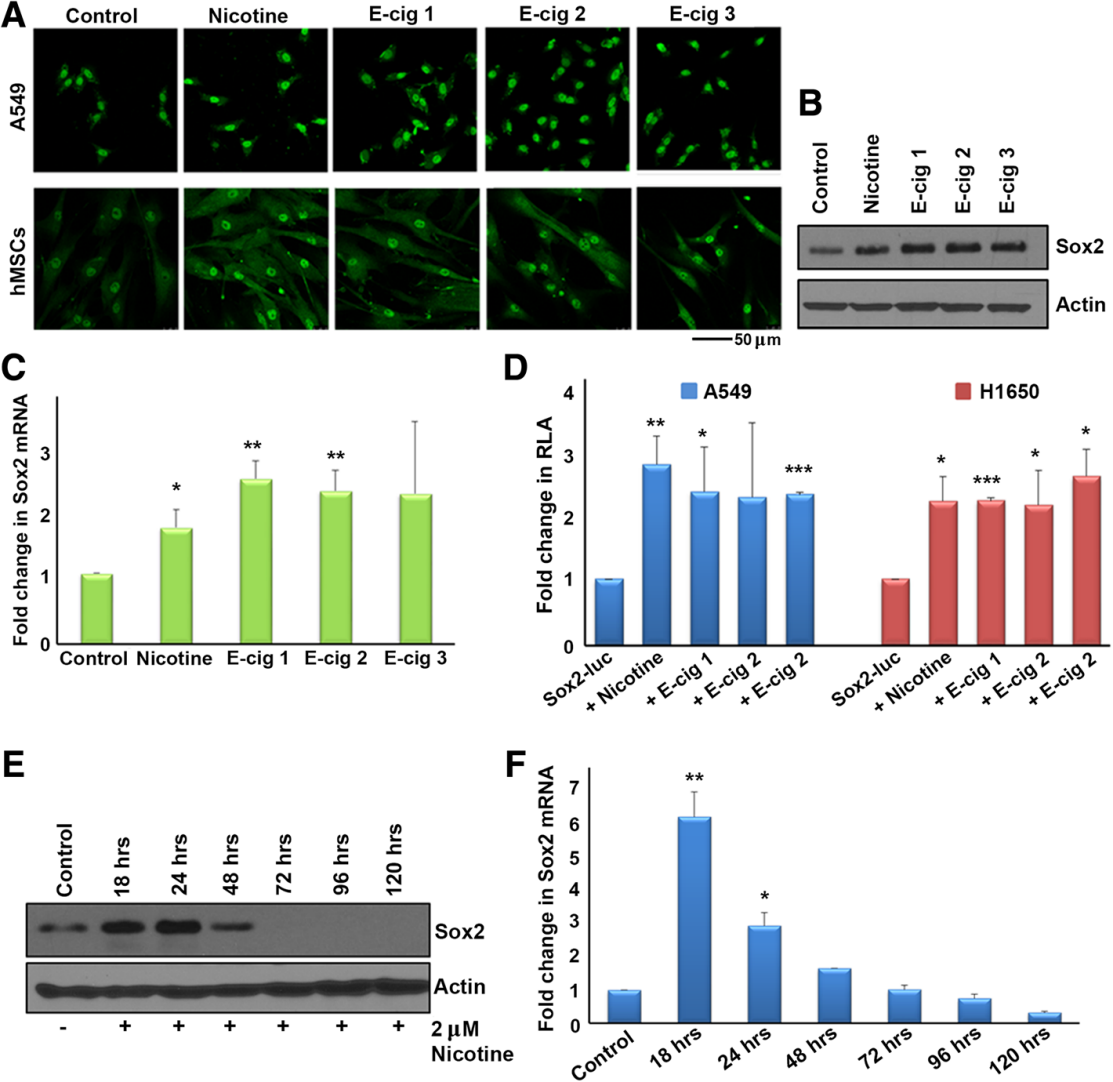
**a** An immunofluorescence experiment showing the induction of Sox2 by nicotine and E-cigarette extracts in A549 cells (top panels) and human mesenchymal stem cells (bottom panel). **b**. The induction of Sox2 by the same agents as seen by western blotting. **c** An RT-PCR experiment shows the induction of Sox2 by nicotine and E-cigarette extracts occurring at the transcriptional level in A549 cells. **d** Treatment of A549 and H1650 cells that are transiently transfected with a Sox2-Luciferase reporter with nicotine and E-cigarette extracts induces promoter activity, confirming the induction of Sox2 at the transcriptional level. **e** A western blot showing the induction of Sox2 protein by nicotine from 18 h to 48 h post treatment **(f**) A RT-PCR experiment showing the induction of Sox2 message at the same time points. The graphical data represented in this figure has ± standard deviation (SD) values derived from three independent experiments. The statistical comparisons between the groups were carried out by unpaired two tailed Student’s t-test

Our lab had reported that the embryonic stem cell transcription factor Sox2 is indispensable for the self-renewal of SP cells from lung adenocarcinomas [27], while Oct4 and Nanog had a relatively lesser role. Further, stimulation with nicotine could enhance the self-renewal of SP cells in a nicotinic acetylcholine receptor dependent manner [16]. We first sought to elucidate whether nicotine enhances self-renewal through induction of Sox2 in A549 and H1650 lung adenocarcinoma cell lines, and whether e-cigarette extracts had similar effects compared to nicotine. Cells were serum starved for 24 h and stimulated with 2 μM nicotine; induction of Sox2 was examined by immunofluorescence microscopy. Nicotine or extracts from each of three brands of e-cigarettes, E-cig 1, E-cig 2, and E-cig 3, could induce Sox2 after 21 h of nicotine stimulation in A549 lung cancer cells. Similar induction was also observed in human mesenchymal stem cells (hMSCs), suggesting that the induction of Sox2 by nicotine is not restricted to cancer cells (Fig. 2a).

Western blot and qRT-PCR experiments confirmed that E-cig 1, E-cig 2, or E-cig 3 could induce Sox2 mRNA and protein expression in A549 cells after 21 h of stimulation, at levels comparable to nicotine (Fig. 2b and c). To further confirm this induction, a Sox2 core promoter luciferase construct (Sox2-luc) was transiently transfected into cells, followed by nicotine and e-cigarette extract stimulation. This construct contained the region -530 bp upstream through + 238 of the transcription start site (TSS) (where TSS = 0) on the Sox2 gene promoter driving the luciferase reporter. Nicotine and each of the three brands of e-cigarette extracts induced Sox2-core-luc activity after 21 h in A549 and H1650 cells (Fig. 2d).

To determine the time course of the nicotine-mediation induction of Sox2, A549 cells were stimulated with nicotine for 18, 24, 48, 72, 96, and 120 h and Sox2 expression was assessed by western blotting and qRT-PCR. 2 μM nicotine could induce Sox2 protein as well as mRNA at 18 and 24 h, an effect that was diminished by 48 h and completely abolished by 72 h (Fig. 2e and f). The disappearance of Sox2 by 72 h of nicotine treatment could be due to the cells acquiring sufficient downstream targets to maintain stemness and may no longer require Sox2. Since we saw the peak induction of Sox2 occurring after 18–24 h of nicotine stimulation, we used 21 h as the time point for the majority of the remaining experiments.

### Nicotine-mediated induction of Sox2 occurs through a nAChR-Yap1-E2F1 axis

**Fig. 3:**
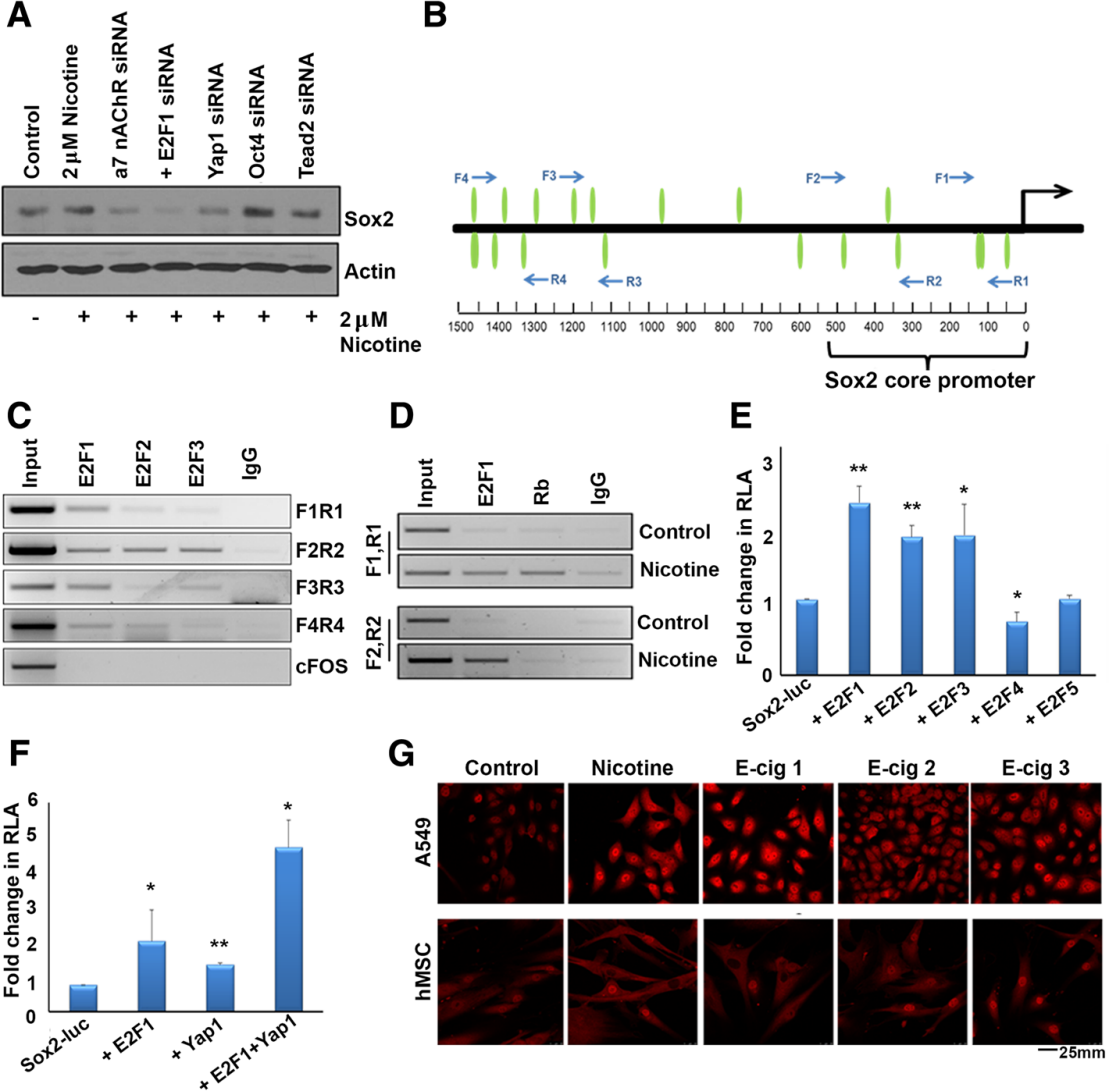
**a** Depletion of E2F1, YAP1 or α7 nAChR by siRNA prevents the nicotine-mediated induction of Sox2. Depletion of Oct4 or TEAD2 did not have any impact on the induction. **b** A schematic showing the location of the E2F binding sites that were tested by ChIP assays; the location of forward and reverse primers for each site are indicated by arrows. **c** A ChIP assay shows the association of E2F1 with all the tested binding sites; E2Fs 2 and 3 were mainly associated with the binding site spanned by primers F2 and R2. There was no E2F associated with c-Fos promoter. **d** Nicotine stimulation induces the association of E2F1 with sites spanned by primers F1R1 and F2R2; surprisingly, Rb could also be detected on site F1R1. **e** A transient transfection experiment conducted on A549 cells showing the induction of Sox2-Luc reported by E2F family members. The data represented is with ± standard deviation (SD) values derived from three independent experiments. The statistical comparison was carried out by unpaired two tailed Student’s t-test. **f** YAP1 and E2F1 can induce the Sox2 promoter, and can show an additive effect in transient transfection experiments. The data represented is with ± standard deviation (SD) values derived from three independent experiments. The statistical comparison was carried out by one-way ANOVA. **g** Nicotine and E-cigarette extracts induce YAP1 levels in A549 cells and human mesenchymal stem cells, as seen by an immunofluorescence experiment

Given our finding that nicotine induces Sox2, and since Sox2 is necessary for the self-renewal of SP cells, we next sought to elucidate the mechanism by which nicotine induces expression of Sox2. Previous studies have shown that nicotine exerts a number of tumor promoting properties such as proliferation, migration, and invasion through the binding to and activation of α7 nAChR, and that the nicotine-mediated activation of these receptors results in the transcriptional activity of E2F1 transcription factor [4, 9, 13, 41]. We have also previously reported that Yes Associated Protein 1 (Yap1), which is a transcriptional co-activator and the major effector of the Hippo signaling pathway, binds to Oct4 embryonic stem cell transcription factor on the Sox2 promoter to regulate both Sox2 expression as well as the stem-like functions of cancer stem-like cells. Induction of Sox2 by Yap1 occurred independent of TEAD2 transcription factor, which is a well-documented binding partner and mediator of Yap1 functions [30]. To evaluate the relative contributions of these proteins to the induction of Sox2, we depleted α7 nAChR, E2F1, Yap1, Oct4, or Tead2 using siRNA in A549 cells, stimulated with nicotine, and conducted western blot experiments. It was found that depletion of α7 nAChR, E2F1, or Yap1 could abrogate nicotine-mediated induction of Sox2 at the protein level (Fig. 3a). To verify that the siRNAs used could reduce the expression of the indicated factors, transient transfection experiments were conducted in untreated A549s cells demonstrating that siRNAs targeting α7 nAChR, E2F1, Yap1, Oct4, Sox2, or TEAD2 effectively reduced their corresponding protein levels, as seen by western blotting (Additional file 1: Figure S1 A-E). This led us to hypothesize that perhaps nicotine-mediated activation of α7 nAChR elevates Yap1’s binding to E2F1 on the Sox2 promoter to induce its expression. Based on our results, we conclude that nicotine mediated activation of α7 nAChR leads to the downstream signaling events resulting in the induction of Sox2 and self-renewal.

### Sox2 gene expression is regulated by E2F1, and nicotine enhances E2F1 binding to Sox2 promoter

To determine whether E2F transcription factors can bind to the Sox2 gene promoter and contribute to its expression, Genomatix MatInspector Analysis Software was used to analyze a 1500 bp region upstream of the Sox2 transcriptional start site for potential E2F binding sites; 19 predicted E2F binding sites were detected on this promoter region (Fig. 3b). Primers were designed spanning four different regions of the Sox2 promoter and chromatin immunoprecipitation assays were carried out to verify if E2Fs bound to the predicted regions. E2F1, and to a lesser extent E2F2 and E2F3, could bind to a region − 104 through –259 bp upstream of TSS, denoted as F1R1, in A549 cells; E2F1, E2F2, and E2F3 could also bind to a region could bind to a region − 266 through − 497 bp upstream of TSS denoted as F2R2; E2F1 and to a lesser extent E2F2 and E2F3 could bind could to a region − 1078 through − 1233 bp upstream of TSS denoted as F3R3; and E2F1 and to some extent E2F2 could bind to a region − 1414 through –1433 bp upstream of TSS denoted as F4R4. An irrelevant IgG antibody was used as a negative control for IP in these experiments, which showed no amplification for any region, and c-Fos was used as a negative promoter control (Fig. 3c). A549 cells were serum starved and treated with nicotine, and ChIP assays were conducted to assess whether the association of E2F1 with the Sox2 promoter was responsive to nicotine stimulation. It was found that nicotine could enhance recruitment of E2F1 to the Sox2 promoter after 18 h of stimulation, and both the F1R1 region − 104 through -259 bp and the F2R2 region − 266 through − 497 bp upstream of TSS (Fig. 3d, bottom lanes).

Given that E2Fs could bind to the Sox2 promoter, transient transfection experiments were conducted to determine whether E2Fs could regulate Sox2 expression; a luciferase reporter driven by a Sox2 core promoter was used for this purpose. It was found that the transcriptionally active E2F family members (E2F1–3) could induce Sox2-core promoter-luciferase activity by 2.3, 1.8, and 1.8-fold respectively, while little or no effect was seen with the inactive E2F4 and E2F5 (0.7 and 1.0 fold respectively) in A549 cells (Fig. 3e). These results show that E2F1 can induce Sox2, and nicotine is possibly inducing Sox2 through this transcription factor.

Since we had previously reported that Yap1 could induce Sox2 expression and here we find that E2F1 also induces Sox2 expression, we next sought to determine whether Yap1 and E2F1 cooperatively induce the Sox2 promoter. Transient transfection experiments showed that Yap1 or E2F1 alone could induce Sox2-core-luc activity by 1.8 and 2.7-fold respectively; when both were transfected together, they showed an 6.2 fold induction of Sox2-core-luc activity, indicating a co-operative effect (Fig. 3f).

### Nicotine and e-cigarette extracts enhance Yap1 expression

Interestingly, we observed that the expression of Yap1 was also enhanced upon nicotine stimulation. Indeed, it has been reported that nicotine could induce Yap1 in certain cell types [42]. Additional immunofluorescence microscopy experiments in A549 cells showed that nicotine and all three brands of e-cigarette extracts induced Yap1 levels after 21 h. Nicotine could induce Yap1 expression in hMSCs to a lesser extent as well, demonstrating that the induction is not specific to NSCLC cells (Fig. 3g).

### Binding of Yap1 to E2F1 is enhanced by nicotine or e-cigarette extracts

**Fig. 4:**
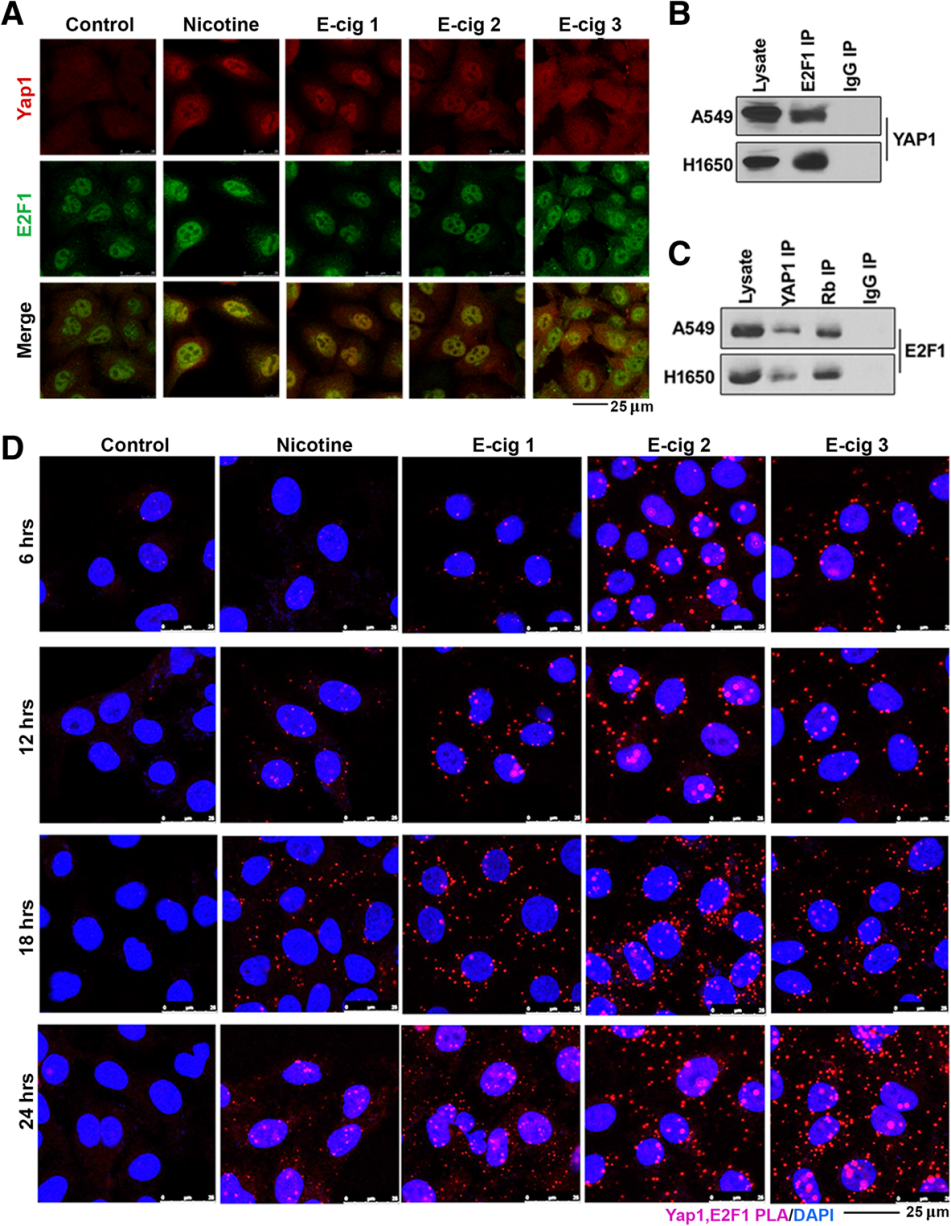
**a** Nicotine and E-cigarette extracts promote the co-localization of E2F1 and YAP1 in A549 cells, as seen by a double immunofluorescence experiment followed by confocal microscopy. **b** An immunoprecipitation-western blot experiment showing the association of YAP1 with E2F1 in A549 and H1650 cells; the immunoprecipitation was conducted by an E2F1 antibody followed by western blotting with a YAP1 antibody. **c** An IP-western blot experiment in the reverse direction, where a YAP1 antibody was used for IP followed by western blotting with an E2F1 antibody, further confirms the association of YAP1 with E2F1. IP with an antibody to Rb was used as a positive control in this experiment. **d** A proximity ligation assay showing the enhanced association of YAP1 with E2F1 in A549 cells upon treatment with nicotine or E-cigarette extracts. The interaction was maximal at 24 h

It has been suggested that Yap1 could co-operate with E2F1-mediated transcription programs in certain cell types [43, 44]. Given that Yap1 can regulate Sox2 expression, and since E2F1 was found to associate with the Sox2 promoter, we next examined whether Yap1, could co-localize or interact with E2F1 and if this was sensitive to nicotine or e-cigarette extract stimulation. Double immunofluorescence experiments showed that Yap1 and E2F1 co-localized in A549 cells; treatment with nicotine or extracts from each of the three e-cigarette brands enhanced this interaction (Fig. 4a). To further confirm the physical interaction of Yap1 and E2F1 proteins, immunoprecipitation-western blot experiments were conducted; these results showed that Yap1 could physically interact with E2F1 in untreated A549 and H1650 cell lines (Fig. 4b and c). Yap1 could be detected in E2F1 immunoprecipitates, by western blotting, and vice versa. An irrelevant IgG was used as a negative control in both cases to establish the specificity of the assay (Fig. 4b and c).

Similarly, proximity ligation assays (PLA) [14, 45–48] were conducted to determine whether Yap1 and E2F1 proteins exist in close proximity within the cell, and at what point in time this occurs. PLA is able to detect proteins within a 40 nm range of one another, which generally indicates a direct physical interaction, and this can be visualized as individual foci by confocal microscopy. These assays showed that Yap1 and E2F1 interacted with one another, and the interaction was enhanced by nicotine as well as extracts from each of the three brands of e-cigarettes. We further find that this effect increased from 6 to 12 to 18 to 24 h of nicotine or e-cigarette extract stimulation, coinciding with the time points at which Sox2 is induced (Fig. 4d).

### Nicotine-mediated induction of Sox2 occurs through Src kinases

**Fig. 5:**
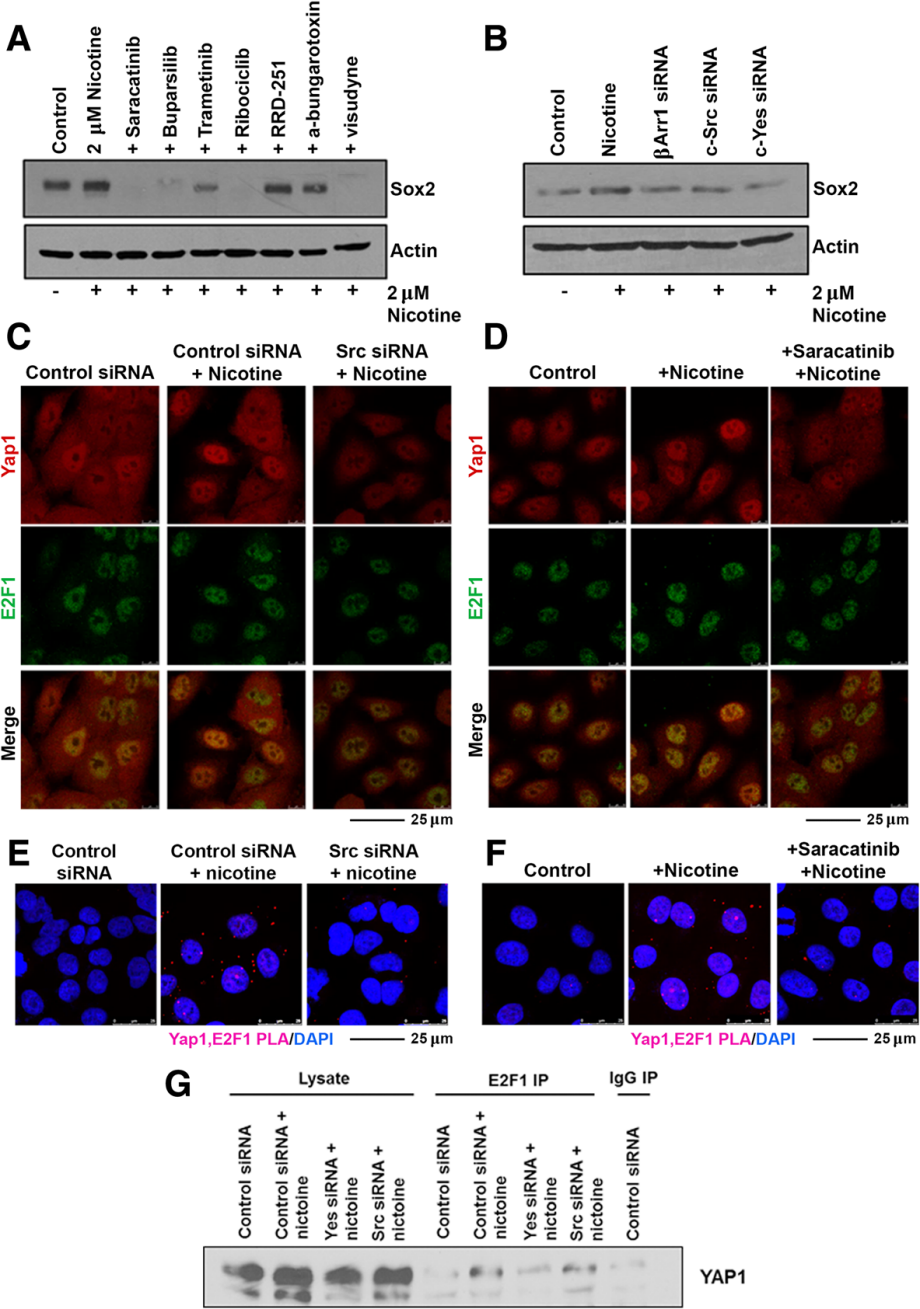
**a** Treatment with selected inhibitors prevents nicotine-mediated induction of Sox2; the Src family kinases appear to be especially vital for the induction. **b** Depletion of Src, Yes or β-arrestin-1 reduces the nicotine-mediated induction of Sox2 in A549 cells (**c**) Treatment with a siRNA to Src reduces YAP1 levels and its interaction with E2F1; similar results were obtained upon treatment with a Src inhibitor (**d**). **e** Proximity ligation assay showing that depletion of Src abrogates the interaction of YAP1 with E2F1; similar results were obtained upon treatment with a Src inhibitor (**f**). **g** An IP-western blot experiment showed that depeltion of Yes and perhaps Src reduces the icotine-mediated interaction of YAP1 with E2F1

The Yap1 protein is known to be oncogenic as it promotes growth while inhibiting apoptosis, and is amplified or overexpressed in a number of cancer types [49–51]. It is named Yes-associated-Protein 1 due to its ability to associate with SH3 domains of Src family tyrosine kinases, which include Src, Yes, and Fyn [49]. Typically in quiescent cells, Yap1 is phosphorylated downstream of the tumor suppressive Hippo pathway, by Lats1 and Lats2 kinases; phosphorylation leads to its sequestration in the cytoplasm by 14–3-3 proteins resulting in proteasomal degradation, thereby preventing its nuclear import [52]. Conversely, Yap1 is phosphorylated by Yes1 in embryonic stem cells, leading to its activation. Multiple studies have now reported that Yap1 can function independent of the Hippo pathway, and it has further been shown that Yap1 is a direct phosphorylation target of Src in a number of cancer cell lines, independent of the canonical Hippo pathway [7, 53, 54]. Our lab has previously reported that when nicotine binds to α7 nAChR, the scaffolding protein β-arrestin-1 (β-arr-1) is recruited and activates Src kinase (p-Src), which mediates a number of downstream pathways, including E2F1 transcriptional activity [13, 35]. We have additionally reported that inhibition of the EGFR pathway including Src and PI3K could strongly inhibit Sox2 expression, thereby suppressing the self-renewal of SP cells; and this occurred in a β-arrestin-1 dependent manner [27, 55]. In this context, we next sought to determine if nicotine-mediated regulation of Sox2 by Yap1 and E2F1 was a result of the upstream activation of Src or Yes kinases. Initial studies were carried out in A549 cells using inhibitors to Src/Yes/Fyn (Saracatinib), PI3K (Buparsilib), MEK1/2 (Trametinib), CDK4/6 (Ribociclib), Rb-Raf interaction (RRD251), α7 nAChR (α-bungarotoxin), and Yap1 (visudyne), to determine which molecules downstream of nAChR facilitate Sox2 induction by nicotine. Cells were treated with inhibitors as described in materials and methods, stimulated with nicotine for 21 h, and Sox2 expression was assessed by western blotting. It was found that treatment with inhibitors to Src/Yes/Fin, PI3K, CDK4/6, or Yap1 could significantly abrogate nicotine-mediated induction of Sox2 (Fig. 5a).

To further confirm whether Src or Yes played a role, we conducted depletion experiments in A549 cells using siRNA targeting β-arr-1, Src, or Yes, following which cells were stimulated with nicotine for 21 h; subsequently, protein levels of Sox2 were assessed by western blotting. Depletion of β-arr-1, Src, or Yes could reduce the induction of Sox2 following nicotine treatment (Fig. 5b). Western blotting was conducted to confirm that siRNA targeting β-arrestin-1, c-Src, or c-Yes1 could deplete the corresponding proteins; it was found that siRNA transfections resulted in reduced levels of protein expression, as expected (Additional file 1: Figure S1).

Double immunofluorescence experiments showed that depletion of Src using siRNA or inhibition using Saracatinib could reduce the co-localization of Yap1 and E2F1 in response to nicotine stimulation after 21 h (Fig. 5c and d); there was a reduction in the levels of Yap1 as well, in both the cases. These results were recapitulated in PLA experiments conducted in the same manner (Fig. 5e and f). These results were supported by an IP-western blot experiment (Fig. 5g). Together, this data suggests that Src family kinases act upstream of Yap1 and E2F1 to regulate induction of Sox2 expression in response to nicotine stimulation.

### Oct4 contributes to nicotine-mediated induction of Sox2

**Fig. 6:**
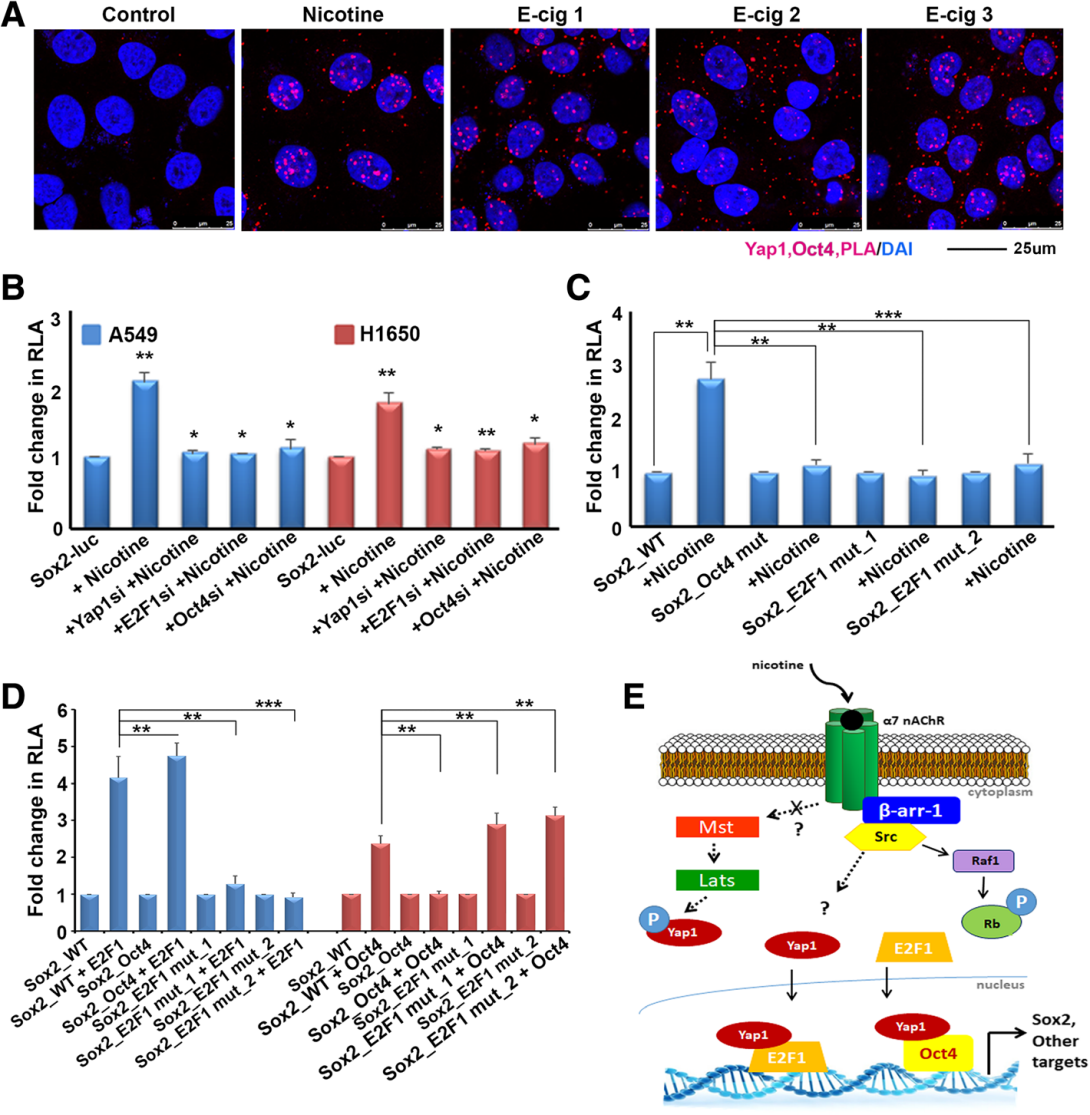
**a** Nicotine can induce the association of Oct4 with YAP1 in A549 cells, as seen by a proximity ligation assay. **b** Depletion of Oct4, E2F1 or YAP1 prevents the nicotine-mediated induction of Sox2-Luc in A549 and H1650 cells. **c** Similarly, mutating Oct4 or E2F binding sites on the Sox2 promoter prevents its induction by nicotine in A549 cells, as seen in a transient transfection experiment. **d** Mutating Oct4 binding sites prevents the induction of Sox2-Luc by Oct4, but the promoter is responsive to E2F1; conversely, mutating E2F binding site 1 or site 2 prevents the induction of the Sox2-Luc reporter by E2F1, but conserves the response to Oct4 in a transient transfection experiment. The graphical data represented in this figure has ± standard deviation (SD) values derived from three independent experiments. The statistical comparisons between the representative groups were carried out by one-way ANOVA to determine the statistical significance. **e** Schematic representing the nicotine mediated upregulation of Sox2. Nicotine binds to the α7 nAChR receptor and activates Src in a βArr1 dependent manner which promotes the binding of to Oct4 and E2F1, resulting in the induction of Sox2

We had previously shown that Yap1 was elevated in cancer stem-like cells from NSCLC and was necessary for their self-renewal and ability to form angiogenic tubules; and these effects of Yap1 were mediated through the induction of Sox2 [30]. Further, depletion of Yap1 resulted in the inability of NSCLC cell lines to form tumors and metastasize in murine orthotopic lung implantation models, and the overexpression of Sox2 could rescue this effect [30]. While this supports an important role for Yap1 in modulation of stem-like functions through the regulation of Sox2, it led us to question whether Yap1 interaction with Oct4 might also play a role in nicotine-mediated induction of Sox2, in addition to Yap1-E2F1 interaction. PLA experiments were conducted to determine whether nicotine or e-cigarette extracts had an effect on the interaction of Yap1 with Oct4 in A549 cells (Fig. 6a). These assays showed that Yap1 and Oct4 existed in close proximity of one another, and the interaction was enhanced by nicotine as well as extracts from each of the three brands of e-cigarettes similar to what we found for Yap1-E2F1 interaction.

We next sought to determine whether Oct4 in addition to E2F1, had a role in nicotine induction of Sox2 itself. This was first assessed by determining whether depletion of Oct4 protein in addition to depletion of E2F1 protein could abrogate nicotine-mediated induction of Sox2, further implicating their roles in this process downstream of Yap1. Transient transfections were conducted in A549 cells where Yap1, E2F1, or Oct4 were depleted using siRNA and transiently transfected using the Sox2-luc reporter, serum starved for 24 h, and stimulated with 2 μM nicotine for 21 h. These experiments demonstrated that depletion of each of the three proteins could abrogate nicotine-mediated induction of Sox2, further supporting their roles in this signaling cascade (Fig. 6b). Since these results demonstrated the inability of nicotine to induce Sox2 expression when Oct4 or E2F1 were depleted, we next sought to determine whether disruption of the binding sites of these transcription factors on the Sox2 promoter had similar effects. Experiments were conducted using A549 cells stably expressing either a wild type Sox2-luc reporter (Sox2_WT), a Sox2-luc reporter with the Oct4 site deleted at a region − 95 through -105 bp upstream of the TSS of the Sox2 promoter to prevent Oct4 binding (Sox2_mutOct4), or each of two clones of a Sox2-luc reporter with seven E2F sites mutated to prevent E2F binding (Sox2_mutE2F1_1 or Sox2_mutE2F1_2). Cells were serum starved and subsequently stimulated with 2 μM nicotine for 21 h. These assays showed that while nicotine could enhance Sox2_WT expression, it could not enhance the expression of the Sox2 reporter in cells lacking an Oct4 binding site or in cells with mutated E2F binding sites, suggesting that the Oct4 site might contribute to nicotine-mediated induction of Sox2-luc activity in addition to the E2F sites (Fig. 6c). To further validate the importance of the Oct4 and E2F binding sites on the Sox2 promoter and assess whether there may be interplay between the two factors, transient transfections were the conducted using each of the stably expressing cell lines to see whether overexpression of E2F1 could still induce Sox2 in cells with altered Oct4 or E2F binding sites, or conversely if Oct4 overexpression could still induce cells with altered Oct4 or E2F binding sites. These experiments demonstrated that when E2F binding sites were mutated on the Sox2 promoter, E2F1 could no longer induce Sox2 but Oct4 could; and conversely when Oct4 sites where deleted on the Sox2 promoter Oct4 could no longer induce Sox2 while E2F1 could (Fig. 6d). Overall, Yap1 seems to have an integral role in the regulation of Sox2 and its induction by nicotine, an effect which is moderated through is transcriptional collaboration with E2F1 and Oct4; however, whether these two transcription factors function independently or together in this context remains elusive.

## Discussion

CSCs represent a subpopulation of tumor cells with increased tumor-initiating capability. They can divide asymmetrically to replenish the heterogenous tumor bulk, and are highly efficient in initiating tumors upon implantation in animal models [22]. CSCs are resistant to various treatment modalities in part due to their enhanced ability to efflux drugs; additional reasons include the fact that they are slower cycling, and they express higher levels of anti-apoptotic proteins. At the same time, complete mechanisms underlying the drug resistance of these cells are not fully understood [22]. Additionally, these cells are thought to remain dormant and facilitate tumor recurrence and metastasis [22, 25]. Not surprisingly, based on these properties of CSCs, efforts are being made to elucidate mechanisms underlying the biology of CSCs in order to target this subpopulation. CSCs have different gene regulatory programs, including epigenetic changes, than the bulk tumor cells; understanding what these differences are, how the programs are regulated, will open up new opportunities for therapeutic targeting.

We have previously reported that nicotine could enhance self-renewal of NSCLC SP cells [16]. The schematic In Fig. 6e represents the proposed mechanism of nicotine mediated induction of Sox2 and possibly stemness [16]. Our earlier studies [12, 13, 35, 56] as well as the current study showed that nicotine binding to the α7 nicotinic acetyl choline receptor recruits β-arr-1 and Src (Fig. 6e). This results in the activation of Yap1, which has been shown to be a target of Src and Src family members [7, 53, 54]. α7 nAChR-mediated activation of Src leads to the phosphorylation of Rb and its dissociation from E2F1, enhancing the transcriptional activity of E2F1 (Fig. 6e) [12, 13, 15, 35, 41, 56]. As mentioned earlier, Yap1 has been found to interact with E2F1 and promote the expression of its downstream targets. Our study suggests a potentially new mechanism by which nicotine induces Sox2 expression in NSCLC cells through Yap1 and its interaction with transcription factors like E2F1 or Oct4 (Fig. 6e)*.* We also find that nicotine induces expression of Yap1 itself, and that the nicotine-mediated induction of Sox2 and Yap1 is not just specific to lung cancer cells but is also observed in human mesenchymal stem cells. One previous report has demonstrated the ability of nicotine to induce Yap1 in esophageal squamous cell carcinoma (ESCC), and this occurred through nAChRs [42]. Interestingly, they find that Yap1 physically interacts with nAChRs and stimulation with nicotine could induce nuclear translocation and activation of Yap1 by disrupting its association with a negative regulatory complex in the cytoplasm composed of α-catenin, β-catenin, and 14–3-3 proteins [42]. The molecular mechanisms regulating this process are not completely understood.

Our prior studies have shown that Yap1 regulates Sox2 through the binding to Oct4 transcription factor, facilitating self-renewal and vascular mimicry [30]. Here we report that E2F1 transcription factor can regulate the Sox2 promoter, and that Yap1 binds to E2F1 likely modulating this effect. Further, we also find that nicotine or e-cigarette extracts can increase the binding of Yap1 to both E2F1 and Oct4. Nicotine has been shown to induce E2F1 transcriptional activity through a sequence of signaling events mediated downstream of nAChRs [35]. Upon nicotine binding, β-arrestin-1 scaffolding protein is recruited to the receptor and activates Src kinase, which subsequently activates Raf-1. Raf-1 then acts to phosphorylate the Rb tumor suppressor protein, which is typically bound to E2F1 during cellular quiescence; but dissociation of hyperphosphorylated Rb from E2F1 allows it to turn on a number of promoters involved in proliferation and survival [57]. We now find that this pathway might contribute to the induction of stemness, by facilitating the expression of Sox2 (Fig. 6e). The downregulation of Sox2 expression after 72 h of nicotine treatment is intriguing; the possibility exists that the cells undergo a transition to a more differentiated state, which might not require the presence of Sox2 by that time point. Alternately, the cells might have acquired sufficient levels of downstream targets of Sox2 to maintain stemness and self-renewal and my not require Sox2 per se by that later time point. It is also likely that the cells might have undergone metabolic changes that allows them to survive in the absence of Sox2.

Our studies also suggest that Yap1 is induced by a non-canonical signaling mechanism in response to nicotine. The Hippo signaling pathway has been demonstrated to have tumor suppressive roles, but is aberrantly altered in multiple cancers including those of the lung [58]. Typically the activation of this pathway by upstream mediators Mst1/2 and Lats1/2 results in the inactivation of Yap1 through its phosphorylation, leading to cytoplasmic sequestration and degradation by 14–3-3 protein [58]. Our results in NSCLC cells suggest that Yap1 is activated through Src and Yes kinases in response to nicotine; the role of the canonical Hippo signaling pathway in the induction remains unclear.

Overall these studies suggest that upon nicotine binding to α7 nAChR, Src is activated and subsequently leads to Yap1 binding to E2F1 and/or Oct4, upregulating Sox2 expression, thereby enhancing self-renewal of CSCs. However, the role of Oct4 in this process is not fully clear. When endogenous expression of Oct4 is knocked down, nicotine could still induce Sox2; in contrast, in cell lines stably expressing a Sox2 promoter containing a mutation of the Oct4 site prevented nicotine-mediated induction of Sox2-luciferase. The molecular basis for the difference in the induction of endogenous Sox2 versus artificially induced Sox2-luciferase remains elusive at this time. It could be that other proteins are forming complexes with Oct4 or E2F1 to regulate Sox2, and these are disrupted by mutation of the Oct4 binding site. It is also possible that post-translational modifications of the proteins involved or histone modifications on Sox2 promoter around the Oct4 binding site play a role.

Our lab had shown that nicotine induces the translocation of β-arrestin-1 scaffolding protein to the nucleus where it binds to E2F1 transcription factors to enhance transcription of E2F target genes [35]. This was found to occur through the formation of an oligomeric complex consisting of β-arrestin-1, E2F1, and p300 histone acetyltransferase proteins which facilitated the acetylation of histones and E2F1, acting to induce transcription of genes involved in proliferation and survival [35]. Our initial experiments show that depletion of β-arrestin-1 reduced endogenous levels of Sox2; this raises the possibility that Yap1 is recruited to a complex with β-arrestin-1 and E2F1 on the Sox2 promoter. Alternately, β-arrestin-1 might be recruiting p300 to E2F1 independent of Yap1. It is additionally worth noting that other E2F family transcription factors may be involved, and their role is worth investigating. These are novel findings that might have a significant impact on our understanding of how nicotine promotes self-renewal of stem-like cells from non-small cell lung cancer. Full elucidation of these mechanisms will shed light on the pathophysiology of smoking-related cancers, and reveal new pathways involved in promotion of CSC populations that can potentially be therapeutically exploited.

## Conclusions

The studies presented here provide compelling evidence that nicotine and components of E-cigarettes can promote the self-renewal of lung adenocarcinoma stem-like cells. This occurs through the mediation of Oct4, Yap1 and E2F1, in response to signaling events from the α7 nAChR. Targeting these pathways and molecules might offer a viable strategy to prevent the self-renewal of stem-like cells and perhaps tumor growth that is promoted by nicotine and E-cigarettes.

## Additional file

Additional file 1: **Figure S1**. (A-E) Transient transfection of siRNA targeting E2F1, Yap1, Oct4, Sox2, or Tead2 to verify that each siRNA could effectively reduce the corresponding protein levels as seen by western blotting. (A) siRNA mediated depletion of E2F1 resulted in reduced expression of E2F1 protein. (B) siRNA mediated depletion of Sox2 or Oct4 resulted in reduced expression of each protein, respectively. (C) siRNA mediated depletion or Yes1 or Tead2 resulted in reduced expression of each protein, respectively. (D) siRNA mediated depletion of Src or beta-Arrestin-1 resulted in reduced expression of each protein, respectively. (E) siRNA mediated depletion of Yap1 resulted in reduced expression of Yap1 protein. (PDF 478 kb)

## Abbreviations

ALDH1: Adehyde dehydrogenase 1
CSC: Cancer stem cells
E2F1: E2 Transcription factor 1
E-Cig: Electronic cigarette
EGFR: Epidermal growth factor receptor
EMT: Epithelial-mesenchymal transition
hMSCs: Human mesenchymal stem cells
MP: Main population cells
NSCLC: Non-small cell lung cancer
Oct4: Octamer binding transcription factor 4
Rb: Retinoblastoma tumor suppressor protein
SCF: Stem cell factor
Sox2: Sex determining region Y box-2 transcription factor
SP cells: Side-population cells
Src: Src proto-oncogene product, non-receptor tyrosine kinase
TSS: Transcription start site
Yap1: Yes associated Protein 1
α7 nAChR: Nicotinic acetylcholine receptor, α7 subunit
