# Supplementary material for: Regulation of Sox2 and stemness by nicotine and electronic-cigarettes in non-small cell lung cancer
Source: Mol Cancer. 2018 Oct 15;17:149. doi: 10.1186/s12943-018-0901-2 (PMC6190543; doi:10.1186/s12943-018-0901-2)
Supplement: Supplementary file 1 — Figure S1. (A-E) Transient transfection of siRNA targeting E2F1, Yap1, Oct4, Sox2, or Tead2 to verify that each siRNA could effectively reduce the corresponding protein levels as seen by western blotting. (A) siRNA mediated depletion of E2F1 resulted in reduced expression of E2F1 protein. (B) siRNA mediated depletion of Sox2 or Oct4 resulted in reduced expression of each protein, respectively. (C) siRNA mediated depletion or Yes1 or Tead2 resulted in reduced expression of each protein, respectively. (D) siRNA mediated depletion of Src or beta-Arrestin-1 resulted in reduced expression of each protein, respectively. (E) siRNA mediated depletion of Yap1 resulted in reduced expression of Yap1 protein. (PDF 478 kb) [file 12943_2018_901_MOESM1_ESM.pdf]

Additional file 1

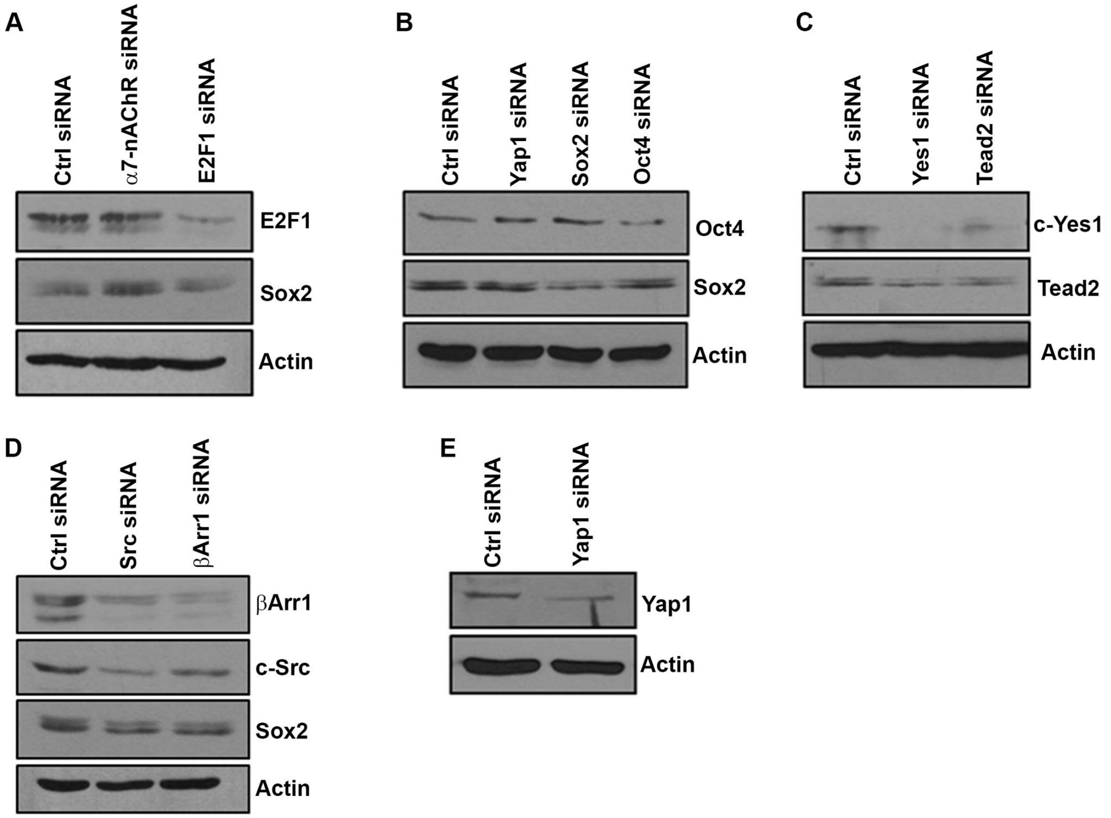

Figure S1 (A- E). Transient transfection of siRNA targeting E2F1, Yap1, Oct4, Sox2, or Tead2 to verify that each siRNA could effectively reduce the corresponding protein levels as seen by western blotting. (A) siRNA mediated depletion of E2F1 resulted in reduced expression of E2F1 protein. (B) siRNA mediated depletion of Sox2 or Oct4 resulted in reduced expression of each protein, respectively. (C) siRNA mediated depletion or Yes1 or Tead2 resulted in reduced expression of each protein, respectively. (D) siRNA mediated depletion of Src or beta-Arrestin-1 resulted in reduced expression of each protein, respectively. (E) siRNA mediated depletion of Yap1 resulted in reduced expression of Yap1 protein.
